# Comparison of the prognostic impact and combination of preoperative inflammation-based and/or nutritional markers in patients with stage II gastric cancer

**DOI:** 10.18632/oncotarget.25486

**Published:** 2018-06-29

**Authors:** Takahiro Toyokawa, Kazuya Muguruma, Tatsuro Tamura, Katsunobu Sakurai, Ryosuke Amano, Naoshi Kubo, Hiroaki Tanaka, Masakazu Yashiro, Kosei Hirakawa, Masaichi Ohira

**Affiliations:** ^1^ Department of Surgical Oncology, Osaka City University Graduate School of Medicine, Abeno-ku, Osaka 545-8585, Japan; ^2^ Department of Gastroenterological Surgery, Osaka City General Hospital, Miyakojima-ku, Osaka 534-0021, Japan

**Keywords:** gastric cancer, gastrectomy, prognostic factor, nutrition, inflammation

## Abstract

**Background:**

The aim of this study was to evaluate and compare the prognostic value of preoperative established inflammation-based and/or nutritional markers, C-reactive protein-to-albumin ratio (CAR), neutrophil-to-lymphocyte ratio, platelet-to-lymphocyte ratio, Prognostic Nutritional Index (PNI), Glasgow Prognostic Score, and prognostic index in patients with stage II gastric cancer. We then developed a new prognostic index based on the results of our investigation.

**Methods:**

This study retrospectively reviewed 240 consecutive patients who underwent R0 resection for stage II gastric cancer. Time-dependent receiver operating characteristic curve analyses were performed to assess discrimination ability and to determine optimal cut-off values. Prognostic factors predicting overall survival (OS) were analyzed using Cox proportional hazards models.

**Results:**

Among inflammation-based and/or nutritional markers, multivariate analyses demonstrated CAR and PNI as independent prognostic factors for OS (hazard ratio (HR) 1.707, 95% confidence interval (CI) 1.016-2.867, *p*=0.044 and HR 0.415, 95%CI 0.234-0.736, *p*=0.003, respectively). CAR-PNI score, constructed as the combination of CAR and PNI, was significantly associated with OS, relapse-free survival and cancer-specific survival (p<0.001 each). Multivariate analysis revealed CAR-PNI score as an independent prognostic factor for OS (HR for CAR-PNI score 1: 2.432, 95%CI 1.155-5.118; HR for CAR-PNI score 2: 4.099, 95%CI 1.835-9.157; *p*=0.002).

**Conclusions:**

CAR and PNI are independent prognostic factors providing superior prediction of survival compared to other inflammation-based and/or nutritional markers. CAR-PNI score offers a novel and promising prognostic indicator for patients with stage II gastric cancer.

## INTRODUCTION

Gastric cancer is the fifth most common malignancy and the third leading cause of cancer-related deaths worldwide [1]. Surgical resection is the mainstay of treatment for non-metastatic gastric cancer, and offers the only chance of cure. Although curative resection provides favorable outcomes for early stage gastric cancer, the prognosis of patients with advanced gastric cancer remains unsatisfactory [2]. Precise prediction of prognosis is crucial to achieving an optimal treatment strategy. The TNM classification, assessed according to the depth of tumor invasion and the extent of lymph node and distant metastasis, is currently recognized as the most reliable and widely available prognostic indicator in clinical practice. However, heterogeneity of clinical outcomes is seen even for tumors at the same stage, suggesting the existence of additional factors influencing outcome. Other biomarkers are thus needed to provide more helpful information to predict individual outcomes for gastric cancer patients.

Recent advances in histochemical and molecular biology have allowed the identification of numerous prognostic factors in various malignancies, but most have proven too complicated, expensive, or troublesome for use in daily clinical practice [3, 4, 5]. On the other hand, a number of studies have focused on the importance of not only tumor-related factors, but also host-related factors in predicting the prognosis of cancer patients. Accumulating evidence has indicated that the systemic inflammatory and nutritional statuses of cancer patients affect prognosis [6, 7]. In recent years, several inflammation-based and nutritional markers have been developed, and many studies have revealed that preoperative values for these markers, such as C-reactive protein-to-albumin ratio (CAR) [8, 9, 10], neutrophil-to-lymphocyte ratio (NLR) [11, 12, 13], platelet-to-lymphocyte ratio (PLR) [14, 15, 16], Prognostic Nutritional Index (PNI) [17, 18, 19], Glasgow Prognostic Score (GPS) [20, 21, 22], and prognostic index (PI) [23, 24, 25], have prognostic value for various cancers, including gastric cancer. These markers commonly offer advantages such as simplicity, reduced invasiveness, low cost, easy reproducibility, and wide availability. However, some controversies remain, such as which markers provide the best prognostic value, optimal cut-off values, the effectiveness of combining markers, and the best markers and cut-offs for different populations by type of cancer, disease stage and type of treatment. Stage II gastric cancer accounts for approximately 20% of R0 resected gastric cancers [26]. Adjuvant chemotherapy has been demonstrated to offer survival benefits for stage II gastric cancer [27, 28], but identification of high- or low-risk patients with stage II gastric cancer may be useful in terms of follow-up and adjuvant treatment. This study therefore aimed to evaluate and compare the prognostic value of preoperative inflammation-based and nutritional markers (CAR, NLR, PLR, PNI, GPS and PI) in patients with stage II gastric cancer after curative resection. In addition, we attempted to construct a new prognostic index based on our results.

## RESULTS

### Discrimination ability and cut-off values for inflammation-based and/or nutritional markers

Areas under the curve (AUCs), cut-off values, sensitivities and specificities of inflammation-based and/or nutritional markers based on the results of time-dependent receiver operating characteristic (ROC) curve analyses are shown in Table 1. According to the cut-off values of 0.03 for CAR, 3.13 for NLR, 188 for PLR, and 49.2 for PNI, 162 patients (67.5%) were classified to the low-CAR group and 78 patients (32.5%) to the high-CAR group, 204 patients (85.0%) were classified to the low-NLR group and 26 patients (15.0%) to the high-NLR group, 193 patients (80.4%) were classified to the low-PLR group and 47 patients (19.6%) to the high-PLR group, and 136 patients (56.7%) were classified to the low-PNI group and 104 patients (43.4%) to the high-PNI group, respectively.

**Table 1:** AUC, cut-off, sensitivity, and specificity

Variables	AUC	Cut-off	Sensitivity (%)	Specificity (%)
CAR	0.641	0.03	54	73
NLR	0.560	3.13	28	88
PLR	0.538	188	28	83
PNI	0.631	49.2	78	49
GPS	0.543	0	24	84
PI	0.541	0	17	92

Abbreviations: AUC, area under the curve; CAR, C-reactive protein-to-albumin ratio; NLR, neutrophil-to-lymphocyte ratio; PLR, platelet-to-lymphocyte ratio; PNI, Prognostic Nutritional Index; GPS, Glasgow Prognostic Score; PI, prognostic index.

### Clinicopathological characteristics

Relationships between clinicopathological characteristics and survival are shown in Table 2. The median age of patients was 64.5 years (interquartile range [IQR], 58-71.3 years), and 168 patients (70.0%) were male. Median body mass index (BMI) was 22.2 kg/m^2^ (IQR, 20.8-24.6 kg/m^2^) and median tumor diameter was 40.0 mm (IQR, 30.0-60.0 mm). The majority of patients were performance status (PS) 0 (83.3%). Operative procedures consisted of total gastrectomy for 72 patients, and partial gastrectomy for 168 patients (proximal gastrectomy in 1 patient, distal gastrectomy in 167 patients). Oral fluoropyrimidines were administered as adjuvant chemotherapy in 178 cases (74.2%) as follows: UFT, 119 cases (66.9%); S-1, 34 cases (19.1%); 5’DFUR, 18 cases (10.1%), and 5-FU, 7 cases (3.9%). Median CAR, NLR, PLR, and PNI were 0.026 (IQR, 0.023-0.054), 1.92 (IQR, 1.36-2.56), 133 (IQR, 101-178), and 48.5 (IQR, 45.0-51.9), respectively. The majority of patients were GPS 0 (82.5%) and PI 0 (90.0%).

**Table 2:** Univariate analyses of prognostic factors for OS of stage II gastric cancer

Variables	5-year OS (%)	Patients		Univariate	
		n	%	HR (95% CI)	*p* value
Total	78.8	240	100		
Age (years)					
≤65	82.2	120	50	1	
>65	75.3	120	50	1.326 (0.818-2.150)	0.253
Sex					
Male	78.7	168	70.0	1	
Female	78.9	72	30.0	1.006 (0.595-1.701)	0.982
BMI (kg/m^2^)					
Low (≤22.2)	82.3	121	50.4	1	
High (>22.2)	75.2	119	49.6	1.326 (0.815-2.157)	0.255
Performance status					
0	81.7	200	83.3	1	
1-3	63.3	40	16.7	2.111 (1.197-3.721)	0.010
Location					
Upper	72.8	57	23.8	1	
Middle	83.4	98	40.8	0.493 (0.267-0.910)	
Lower	78.1	83	34.6	0.797 (0.444-1.433)	
Whole	50.0	2	0.8	1.223 (0.164-9.102)	0.668
Macroscopic type					
Type 0-2	83.9	153	63.8	1	
Type 3-5	69.8	87	36.2	2.122 (1.309-3.440)	0.002
Operative procedure					
Partial gastrectomy	80.8	168	70.0	1	
Total gastrectomy	73.7	72	30.0	1.400 (0.847-2.313)	0.189
Histology					
Differentiated	80.1	108	45.0	1	
Undifferentiated	77.7	132	55.0	0.913 (0.562-1.484)	0.714
Lymphatic invasion					
Absent	91.6	60	25.0	1	
Present	74.4	180	75.0	3.864 (1.669-8.947)	0.002
Venous invasion					
Absent	81.0	192	80.0	1	
Present	69.1	48	20.0	1.762 (1.023-3.035)	0.041
TNM sub-stage					
IIA	86.2	111	46.3	1	
IIB	72.3	129	53.7	1.665 (1.002-2.768)	0.049
Tumor size (mm)					
≤40	79.2	126	52.5	1	
>40	78.1	114	47.5	0.985 (0.608-1.598)	0.952
Adjuvant chemotherapy					
Absent	73.5	62	25.8	1	
Present	80.6	178	74.2	0.850 (0.494-1.462)	0.558
CAR					
Low (≤0.03)	85.5	162	67.5	1	
High (>0.03)	64.7	78	32.5	2.161 (1.332-3.507)	0.002
NLR					
Low (≤3.13)	82.0	204	85.0	1	
High (>3.13)	60.7	36	15.0	2.271 (1.306-3.950)	0.004
PLR					
Low (≤188)	81.0	193	80.4	1	
High (>188)	69.5	47	19.6	1.676 (0.974-2.883)	0.062
PNI					
Low (≤49.2)	70.6	136	56.7	1	
High (>49.2)	89.3	104	43.3	0.381 (0.219-0.662)	0.001
GPS					
0	80.5	198	82.5	1	
1/2	70.5	42	17.5	1.457 (0.808-2.630)	0.211
PI					
0	80.2	216	90.0	1	
1/2	64.5	24	10.0	1.416 (0.676-2.968)	0.357

Abbreviations: BMI, body mass index; PS, performance status; TNM, tumor-node-metastasis; CAR, C-reactive protein-to-albumin ratio; NLR, neutrophil-to-lymphocyte ratio; PLR, platelet-to-lymphocyte ratio; PNI, Prognostic Nutritional Index; GPS, Glasgow Prognostic Score; PI, prognostic index.

### Survival

Median follow-up for survivors was 100.5 months (IQR, 70.0-136.8 months). Six patients were lost to follow-up, with the shortest follow-up period for survivors being 11 months. Recurrence was observed in 50 cases, with a median duration to recurrence of 17 months (IQR, 9.0-42.0 months). A total of 66 deaths were identified.

**Figure 1:**
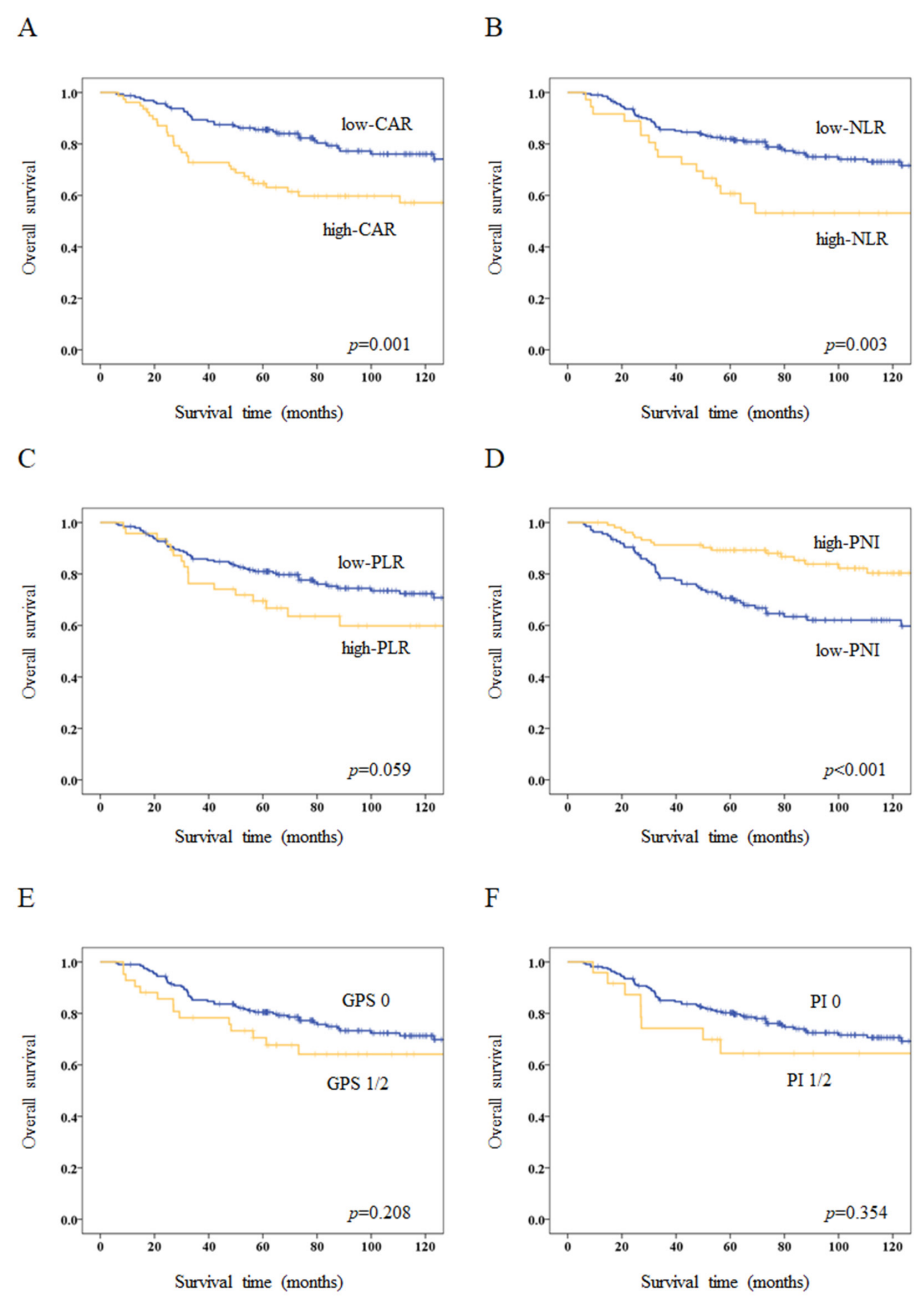
Kaplan–Meier survival curves for overall survival (OS) according to inflammation-based and/or nutritional markers **(A)** Five-year OS rates are 85.5% in the low-CAR group and 64.7% in the high-CAR group (*p*=0.001). **(B)** Five-year OS rates are 82.0% in the low-NLR group and 60.7% in the high-NLR group (*p*=0.003). **(C)** Five-year OS rates are 81.0% in the low-PLR group and 69.5% in the high-PLR group (*p*=0.059). **(D)** Five-year OS rates are 70.6% in the low-PNI group and 89.3% in the high-PNI group (*p*<0.001). **(E)** Five-year OS rates are 80.5% in the GPS 0 group and 70.5% in the GPS 1/2 group (*p*=0.208). **(F)** Five-year OS rates are 80.2% in the PI 0 group and 64.5% in the PI 1/2 group (*p*=0.354).

The 5-year OS rate for the entire study population was 78.8%. Kaplan–Meier survival curves comparing OS between two groups based on each inflammation-based and/or nutritional marker are shown in Figure 1A–1F. OS rates were significantly lower in the high-CAR (*p*=0.001), high-NLR (*p*=0.003), and low-PNI (*p*<0.001) groups.

### Prognostic factors for OS

Results of uni- and multivariate analyses for OS are summarized in Tables 2 and 3. Univariate analyses identified PS, macroscopic type, lymphatic invasion, venous invasion, TNM sub-stage, CAR, NLR, and PNI as significantly associated with OS. Among inflammation-based and/or nutritional markers, multivariate analyses demonstrated CAR and PNI as independent prognostic factors for OS (hazard ratio (HR) 1.707, 95% confidence interval (CI) 1.016-2.867, *p*=0.044 and HR 0.415, 95%CI 0.234-0.736, *p*=0.003, respectively).

**Table 3:** Multivariate analyses of prognostic factors for OS of stage II gastric cancer

Variables	Analysis with CAR		Analysis with NLR		Analysis with PLR		Analysis with PNI	
	HR (95%CI)	*p* value	HR (95%CI)	*p* value	HR (95%CI)	*p* value	HR (95%CI)	*p* value
Performance status (0 vs 1-3)	1.793 (0.981-3.278)	0.058	2.141 (1.207-3.796)	0.009	2.190 (1.235-3.881)	0.007	1.852 (1.037-3.307)	0.037
Macroscopic type (0-2 vs 3-5)	1.742 (1.062-2.858)	0.028	1.809 (1.101-2.971)	0.019	1.981 (1.205-3.254)	0.007	2.006 (1.226-3.284)	0.006
Lymphatic invasion (absent vs present)	3.224 (1.378-7.542)	0.007	3.163 (1.345-7.438)	0.008	3.132 (1.333-7.359)	0.009	3.131 (1.334-7.348)	0.009
Venous invasion (absent vs present)	1.416 (0.813-2.464)	0.219	1.345 (0.760-2.382)	0.309	1.520 (0.874-2.643)	0.138	1.555 (0.894-2.705)	0.118
TNM sub-stage (IIA vs IIB)	1.585 (0.939-2.674)	0.085	1.439 (0.852-2.430)	0.174	1.490 (0.887-2.504)	0.132	1.292 (0.764-2.184)	0.340
CAR (≤0.03 vs >0.03)	1.707 (1.016-2.867)	0.044						
NLR (≤3.13 vs >3.13)			1.621 (0.905-2.904)	0.105				
PLR (≤188 vs >188)					1.717 (0.989-2.981)	0.055		
PNI (≤49.2 vs >49.2)							0.415 (0.234-0.736)	0.003

Abbreviations: TNM, tumor-node-metastasis; CAR, C-reactive protein-to-albumin ratio; NLR, neutrophil-to-lymphocyte ratio; PLR, platelet-to-lymphocyte ratio; PNI, Prognostic Nutritional Index.

### New prognostic index

**Figure 2:**
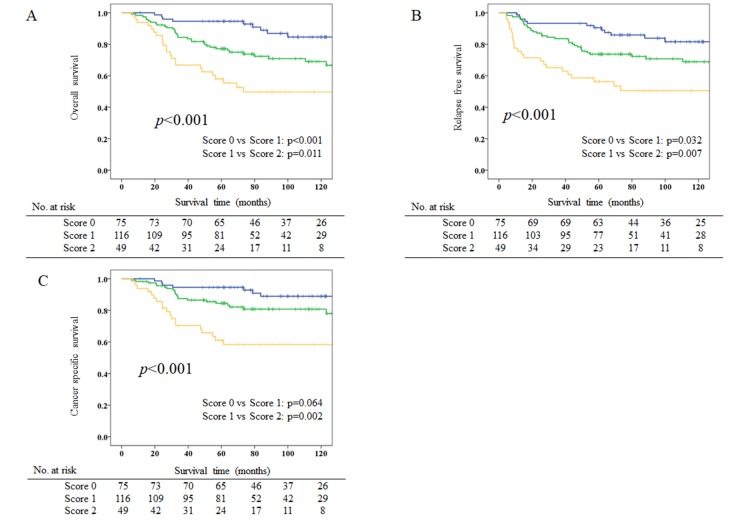
Kaplan–Meier survival curves for overall survival (OS), relapse-free survival (RFS), and cancer-specific survival (CSS) according to CAR-PNI scores **(A)** Five-year OS rates are 94.6%, 77.1% and 58.0% for CAR-PNI score 0, 1, and 2 groups, respectively (*p*<0.001). **(B)** Five-year RFS rates are 90.5%, 73.7% and 56.3% in CAR-PNI score 0, 1, and 2 groups, respectively (*p*<0.001). **(C)** Five-year CSS rates are 94.6%, 84.5% and 61.2% in CAR-PNI score 0, 1, and 2 groups, respectively (*p*<0.001).

According to the results of multivariate analyses, we constructed CAR-PNI score as a new prognostic index, as follows: CAR-PNI score 2, both high-CAR and low-PNI; CAR-PNI score 1, either high-CAR or low-PNI, but not both; and CAR-PNI score 0, neither abnormality. The prognostic value of CAR-PNI score was then evaluated. CAR-PNI scores were 0 for 75 patients (31.3%), 1 for 116 patients (48.3%), and 2 for 49 patients (20.4%). The AUC of CAR-PNI score for predicting 5-year OS was 0.706. The association between CAR-PNI score and clinicopathological characteristics of patients with stage II gastric cancer is demonstrated in Table 4. Higher CAR-PNI score was significantly associated with higher age (*p*=0.001), poorer PS (*p*=0.002), larger tumor diameter (*p*=0.013), higher recurrence rate (*p*=0.002), and other inflammation-based and/or nutritional markers (*p*<0.001 each). Kaplan–Meier survival curves for OS, relapse-free survival (RFS) and cancer-specific survival (CSS) according to CAR-PNI score are shown in Figure 2A–2C. Five-year OS, RFS, and CSS rates for the CAR-PNI score 0, 1, and 2 groups were 94.6%, 77.1%, and 58.0% (*p*<0.001), 90.5%, 73.7%, and 56.3% (*p*<0.001), and 94.6%, 84.5%, and 61.2% (*p*<0.001), respectively. CAR-PNI score allowed clear classification of patients into three groups for each of OS, RFS and CSS. Multivariate analysis revealed CAR-PNI score as an independent prognostic factor for OS (HR for CAR-PNI score 1: 2.432, 95%CI 1.155-5.118 and HR for CAR-PNI score 2: 4.099, 95%CI 1.835-9.157; *p*=0.002) (Table 5).

**Table 4:** Correlation of the CAR-PNI score and clinicopathological characteristics of patients

Variables	CAR-PNI score 0		CAR-PNI score 1		CAR-PNI score 2		*p* value
	n	%	n	%	n	%	
Age (years)							
≤65	51	68.0	61	52.6	16	32.7	
>65	24	32.0	55	47.4	33	67.3	0.001
Sex							
Male	56	74.7	79	68.1	33	67.3	
Female	19	25.3	37	31.9	16	32.7	0.565
BMI (kg/m^2^)							
Low (≤22.2)	32	42.7	64	55.2	25	51.0	
High (>22.2)	43	57.3	52	44.8	24	49.0	0.239
Performance status							
0	71	94.7	94	81.0	35	71.4	
1-3	4	5.3	22	19.0	14	28.6	0.002
Location							
Upper	16	21.3	23	19.8	18	36.7	
Middle	34	45.3	50	43.1	14	28.6	
Lower	25	33.3	41	35.3	17	34.7	
Whole	0	0	2	1.7	0	0	0.178
Macroscopic type							
Type 0-2	51	68.0	76	65.5	26	53.1	
Type 3-5	24	32.0	40	34.5	23	46.9	0.205
Operative procedure							
Partial gastrectomy	57	76.0	85	73.3	26	53.1	
Total gastrectomy	18	34.0	31	26.7	23	46.9	0.014
Histology							
Differentiated	31	41.3	51	44.0	26	53.1	
Undifferentiated	44	58.7	65	56.0	23	46.9	0.418
Lymphatic invasion							
Absent	21	28.0	30	25.9	9	18.4	
Present	54	72.0	86	74.1	40	81.6	0.459
Venous invasion							
Absent	61	81.3	94	81.0	37	75.5	
Present	14	18.7	22	19.0	12	24.5	0.678
TNM substage							
IIA	39	52.0	53	45.7	19	38.8	
IIB	36	48.0	63	54.3	30	61.2	0.348
Tumor size (mm)							
≤40	46	61.3	63	54.3	17	34.7	
>40	29	38.7	53	45.7	32	65.3	0.013
Adjuvant chemotherapy							
Absent	18	24.0	29	25.0	15	30.6	
Present	57	76.0	87	75.0	34	69.4	0.685
NLR							
Low (≤3.13)	74	98.7	99	85.3	31	63.3	
High (>3.13)	1	1.3	17	14.7	18	36.7	<0.001
PLR							
Low (≤188)	72	96.0	90	77.6	31	63.3	
High (>188)	3	4.0	26	22.4	18	36.7	<0.001
GPS							
0	75	100	100	86.2	23	46.9	
1/2	0	0	16	13.8	26	53.1	<0.001
PI							
0	75	100	106	91.4	35	71.4	
1/2	0	0	10	8.6	14	28.6	<0.001
Recurrence							
Absent	65	86.7	95	81.9	30	61.2	
Present	10	13.4	21	18.1	19	38.8	0.002

Abbreviations: BMI, body mass index; PS, performance status; TNM, tumor-node-metastasis; CAR, C-reactive protein-to-albumin ratio; NLR, neutrophil-to-lymphocyte ratio; PLR, platelet-to-lymphocyte ratio; PNI, Prognostic Nutritional Index; GPS, Glasgow Prognostic Score; PI, prognostic index.

**Table 5:** Multivariate analysis of prognostic factors for OS of stage II gastric cancer

Variables	HR (95% CI)	*p* value
Performance status		
0	1	
1-3	1.546 (0.852-2.803)	0.852
Macroscopic type		
0-2	1	
3-5	1.809 (1.109-2.953)	0.018
Lymphatic invasion		
Absent	1	
Present	3.048 (1.302-7.138)	0.010
Venous invasion		
Absent	1	
Present	1.449 (0.835-2.515)	0.188
TNM sub-stage		
IIA	1	
IIB	1.421 (0.847-2.386)	0.183
CAR-PNI score		
0	1	
1	2.432 (1.155-5.118)	0.019
2	4.099 (1.835-9.157)	0.001

*TNM*, tumor-node-metastasis; *CAR*, C-reactive protein-to-albumin ratio; *PNI*, Prognostic Nutritional Index.

### Recurrence patterns and causes of death

Recurrence patterns and causes of death according to CAR, PNI, and CAR-PNI score are shown in Tables 6 and 7. The high-CAR group revealed a significantly higher frequency of hematogenous recurrence (*p*=0.030), whereas the low-PNI group revealed a significantly higher frequency of peritoneal recurrence (*p*=0.041) as the initial recurrence. The CAR-PNI score 2 group revealed a significantly higher frequency of hematogenous recurrence (*p*=0.005). The proportion of patients who died of primary disease was significantly higher in the high-CAR (*p*=0.020), low-PNI (*p*=0.001), and higher CAR-PNI score groups (*p*<0.001).

**Table 6:** Recurrence patterns

	CAR		*p* value	PNI		*p* value	CAR-PNI score			*p* value
	Low (n=162)	High (n=78)		Low (n=136)	High (n=104)		0 (n=75)	1 (n=116)	2 (n=49)	
Hematogenous	8 (4.9%)	10 (12.8%)	0.030	13 (9.6%)	5 (4.8%)	0.166	4 (5.3%)	5 (4.3%)	9 (18.4%)	0.005
Lymph nodes	8 (4.9%)	6 (7.7%)	0.394	9 (6.6%)	5 (4.8%)	0.553	4 (5.3%)	5 (4.3%)	5 (10.2%)	0.328
Peritoneum	15 (9.3%)	7 (9.0%)	0.943	17 (12.5%)	5 (4.8%)	0.041	4 (5.3%)	12 (10.3%)	6 (12.2%)	0.354

**Table 7:** Causes of death

	CAR		*p* value	PNI		*p* value	CAR-PNI score			*p* value
	Low (n=162)	High (n=78)		Low (n=136)	High (n=104)		0 (n=75)	1 (n=116)	2 (n=49)	
Total	35 (21.6%)	31 (39.7%)		49 (36.0%)	17 (16.3%)		9 (12.0%)	34 (29.3%)	23 (46.9%)	
Primary disease	25 (15.4%)	22 (28.2%)	0.020	37 (27.2%)	10 (9.6%)	0.001	7 (9.3%)	21 (18.1%)	19 (38.8%)	<0.001
Other disease	7 (4.3%)	6 (7.7%)	0.280	9 (6.6%)	4 (3.8%)	0.347	1 (1.3%)	9 (7.8%)	3 (6.1%)	0.155
Other cancer	3 (1.9%)	3 (3.8%)	0.354	3 (2.2%)	3 (2.9%)	0.739	1 (1.3%)	4 (3.4%)	1 (2.0%)	0.641

## DISCUSSION

This study of 240 patients with stage II gastric cancer found that CAR and PNI represent independent prognostic factors for OS, and were superior to other inflammation-based markers in terms of predictive ability after curative resection. We also developed a novel marker of both inflammation and nutrition (CAR-PNI score) that provided better prognostic value than either CAR or PNI alone. Notably, this novel index is estimated using only three serum markers that are already routinely measured in daily clinical practice. To the best of our knowledge, this is the first report to examine the usefulness and optimal combination of inflammation-based and/or nutritional markers as a prognostic factor.

Impaired nutritional status is reportedly associated with poor prognosis in various cancer patients [7]. PNI, which offers an assessment of nutritional condition, has been widely investigated for associations with prognosis in cancer patients, due to its simplicity and ease of use. Several recent studies have demonstrated lower PNI as significantly associated with advanced stage, offering independent prognostic value in gastric cancer patients. However, cut-off values and results of subgroup analyses according to tumor stage have differed between studies [17, 18, 19, 30, 31]. For instance, Sun et al. [31] reported lower PNI as significantly associated with poorer OS in stage II and III, but not stage I and IV, whereas Migita et al. [17] reported lower PNI as significantly associated with poorer OS in stage I and III, but not in stage II and IV gastric cancer. Such discrepancies may be attributed to differences in the proportion of patients in each stage among studies, suggesting that cut-offs and prognostic values should be evaluated based on tumor stage. For this reason, we focused solely on patients with stage II gastric cancer as subjects in the present study. Impaired nutritional status in gastric cancer patients is primarily caused by reduced food intake due to physical obstruction by the tumor and the increased metabolic rate of the tumor. Lower PNI in stage II gastric cancer may thus reflect increased energy consumption by the entire tumor associated with tumor aggressiveness that is not indicated by TNM stage, which might lead to PNI being an independent prognostic factor.

NLR, PLR, GPS, and PI are well known inflammation-based markers, and have been reported as prognostic factors for various malignancies [11, 13, 15, 20, 22, 24]. CAR is calculated from serum albumin and CRP levels and was first developed to predict outcomes in patients with acute medical admissions, and has recently gained attention as an inflammation-based marker for predicting outcomes in cancer patients [8, 10, 32, 33]. Several studies have demonstrated that CAR represents an independent prognostic factor with superior prognostic ability compared to other inflammation-based markers in pancreatic cancer [33], ovarian cancer [34], esophageal squamous cell carcinoma [35], and gastric cancer [9]. Consistent with previous studies, the present study revealed CAR as an independent prognostic factor when the cut-off was defined as 0.03, and ROC analyses indicated that CAR had a similar AUC to PNI, and a higher AUC than NLR, PLR, GPS, or PI in patients with stage II gastric cancer.

The present study demonstrated that the CAR-PNI score can serve as a better predictor of survival in patients with stage II gastric cancer than either the CAR or PNI alone. A higher CAR-PNI score was significantly associated with older age, worse PS, other inflammation markers, and larger tumor diameter, which may reflect not only the poorer status of patients, but also an aggressive tumor phenotype. Although the exact mechanisms underlying the prognostic implications of markers for systemic inflammation, immunological and nutritional status of patients have yet to be elucidated, these statuses have repeatedly been associated with prognosis in cancer patients. The CAR-PNI score uses only three key serum markers: serum albumin concentration, serum CRP concentration, and total peripheral lymphocyte count. These three markers have been reported as independent prognostic factors individually in various cancers [36, 37, 38]. As a consequence of cross-linkage of albumin, CRP and lymphocyte count, the CAR-PNI score serves as an index to comprehensively evaluate systemic inflammatory, immunological, and nutritional status, potentially providing more precise and informative prognostic value.

Stage II gastric cancer patients have shown acceptable long-term outcomes thanks to advances in adjuvant chemotherapy, but our results suggest that a population of patients with stage II gastric cancer needing more intensive treatment can be identified. In the present study, CAR-PNI score was able to clearly classify stage II gastric cancer patients into three groups in terms of prognosis. The 5-year OS rate in the CAR-PNI score 0 group without adjuvant chemotherapy was 94.1%, better than the 84.2% for stage II gastric cancer patients who underwent adjuvant chemotherapy in the ACTS-GC trial [27]. On the other hand, the 5-year OS rate in the CAR-PNI score 2 group with adjuvant chemotherapy was 57.4%, resembling the 57.3% in stage III gastric cancer patients without adjuvant chemotherapy reported in the ACTS-GC trial [27]. Finally, the CAR-PNI score 2 group showed significant associations with recurrence, poorer RFS, and poorer CSS, suggesting that the preoperative systemic inflammation and poor nutritional status estimated by the CAR-PNI score may be associated with the growth of micrometastases and residual cancer cells. These findings suggest that a shorter period of adjuvant chemotherapy may be allowable for patients with CAR-PNI score 0, while more intensive adjuvant treatment may be warranted for patients with CAR-PNI score 2 in stage II gastric cancer, as in stage III. Although TNM stage is the mainstay for determining adjuvant therapy in gastric cancer treatment, CAR-PNI score could provide complementary information for clinicians in determining adjuvant chemotherapy for patients with stage II gastric cancer.

The prognostic values of combinations of inflammation-based and/or nutritional markers have not yet been verified. Although our findings suggested CAR-PNI score as a promising prognostic factor, optimal cut-offs and prognostic impacts of CAR and PNI can be expected to differ according to the study population. Indeed, although we attempted the same analyses for patients with stage III gastric cancer, the results were different (data not shown). Further studies are thus needed to identify the optimal combinations of inflammation-based and/or nutritional markers for specific populations, such as tumor stage.

Numerous studies have tried to verify the usefulness of nutritional intervention in perioperative management for various cancers. However, not only have the effects of perioperative nutritional intervention on long-term outcomes in cancer patients not been confirmed, but also few studies have shown correlations between improvement of inflammation and nutritional markers and prognosis of cancer patients [39, 40]. Furthermore, optimal indexes for perioperative nutritional management have not yet been established. CAR, PNI, or CAR-PNI scores may be useful indicators to select patients needing nutritional intervention and to assess nutritional management in patients with stage II gastric cancer.

Some potential limitations should be recognized in the present study. First, this was a retrospective study conducted using data from a single institution. Second, factors that could potentially affect inflammation-based and/or nutritional markers, such as comorbidities and medications, were not controlled for in this study. Third, our study showed heterogeneity in the adjuvant chemotherapies for patients with stage II gastric cancer, representing a possible confounder. However, in subgroup analysis according to the presence or absence of adjuvant chemotherapy, CAR-PNI score was significantly associated with OS in both subgroups (Supplementary Figure 1). Within these limitations, the present findings suggest that CAR-PNI score can help clinicians identify patients with stage II gastric cancer at high risk of recurrence, allowing the provision of intensive adjuvant chemotherapy and closer follow-up. Large-scale prospective validation studies are needed to confirm our findings.

In conclusion, our study demonstrated that PNI and CAR represent independent prognostic factors with superior predictive ability for survival compared to the established inflammation-based and nutritional markers of NLR, PLR, GPS, and PI. In addition, the CAR-PNI score estimated by combining PNI and CAR offers an even better prognostic indicator, and may help provide individualized treatment for patients with stage II gastric cancer.

## PATIENTS AND METHODS

The clinical data of consecutive patients who underwent R0 resection for primary gastric cancer at Osaka City University Hospital (Osaka, Japan) between January 1997 and December 2012 were retrospectively reviewed. All patients had been diagnosed with adenocarcinoma, confirmed as stage II on postoperative pathological examination. We excluded 14 patients who had received neoadjuvant chemotherapy, 12 patients with concomitant malignancies, 3 patients who had died from postoperative complications, and 13 patients for whom the entire set of preoperative laboratory data was not available. Ultimately, 240 patients were included in this study. This retrospective study was approved by the ethics committee at our institution and was conducted in accordance with the principles of the Declaration of Helsinki.

Blood samples were routinely obtained within 1 week before operation. CAR was calculated by dividing the serum C-reactive protein (CRP) level (mg/dl) by the serum albumin level (g/dl). NLR was calculated by dividing the neutrophil count by the lymphocyte count. PLR was calculated by dividing the platelet count by the lymphocyte count. PNI was calculated as 10 × serum albumin level (g/dl) + 0.005 × total peripheral lymphocyte count (per mm^3^). GPS was scored as follows: GPS 2, both CRP >1.0 mg/dl and albumin <3.5 g/dl; GPS 1, either CRP >1.0 mg/dl or albumin <3.5 g/dl, but not both; and GPS 0, neither abnormality. PI was determined as follows: PI 2, both CRP >1.0 mg/dl and white cell count >11 ×10^9^/l; PI 1, either CRP >1.0 mg/dl or white cell count >11 ×10^9^/l, but not both; and PI 0, neither abnormality.

Clinical variables such as age, sex, BMI, Eastern Cooperative Oncology Group performance status (PS), tumor location, macroscopic type, operative procedure, histology, lymphatic invasion, venous invasion, TNM sub-stage, tumor size, and adjuvant chemotherapy were evaluated. Tumors were staged according to the third English edition of the Japanese classification of gastric carcinoma [29]. Median values for age, BMI and tumor size were used as cut-off values. To determine cut-off values for CAR, NLR, PLR, and PNI, time-dependent ROC curve analyses for 5-year overall survival (OS) as the endpoint were calculated, and maximal Youden indices were determined. All patients were classified into two groups based on these cut-off values.

Distal, proximal, or total gastrectomy was performed according to tumor size, location, and status of the resection margins. All surgical procedures were performed by a single team of oncology surgeons specializing in the upper gastrointestinal tract. Adjuvant chemotherapy with oral fluoropyrimidines (5-fluorouracil, uracil-tegafur, doxifluridine, or S-1) was undertaken after obtaining informed consent from the patient. Follow-up was performed every 4 months for the initial 2 years, every 6 months for the next 3 years, and annually thereafter. On a semiannual basis or on suspicion of recurrence, a clinical history was taken, and a physical examination, routine blood tests, measurements of tumor markers, and enhanced abdominal computed tomography were performed. Recurrence was diagnosed according to the findings of these scheduled examinations. If the patient had not visited the hospital, follow-up information was obtained from telephone calls to the patient, family members, or referring physician.

### Statistical analysis

OS, RFS, and CSS were calculated from the date of operation to the date of last follow-up or death, to the date of confirmation of recurrence or death, and to the date of last follow-up or death due to gastric cancer, respectively. Survival rates were calculated using Kaplan–Meier methods, and survival curves were compared using the log-rank test. Uni- and multivariate analyses for OS was conducted with Cox proportional hazards models. To compare the prognostic value of each inflammation-based and/or nutritional marker, multivariate analyses were performed with the inclusion of variables showing values of *p*<0.1 on univariate analysis and each inflammation-based and/or nutritional marker with *p*<0.1, respectively, because NLR, PLR, and PNI include the lymphocyte count, CAR, PNI and GPS include the albumin level, and CAR, GPS and PI include the CRP level in the scoring systems. HRs and 95%CIs were calculated. Values of *p*<0.05 were considered significant. All statistical analyses were performed using SPSS software (SPSS, Chicago, IL), with the exception of time-dependent ROC curve-analyses, which were performed using R-project software, version 3.3.0.

## SUPPLEMENTARY MATERIALS FIGURES



## Abbreviations

CAR: C-reactive protein-to-albumin ratio
NLR: neutrophil-to-lymphocyte ratio
PLR: platelet-to-lymphocyte ratio
PNI: Prognostic Nutritional Index
GPS: Glasgow Prognostic Score
PI: prognostic index
AUC: area under the curve
ROC: receiver operating characteristic
IQR: interquartile range
BMI: body mass index
PS: Eastern Cooperative Oncology Group performance status
OS: overall survival
HR: hazard ratio
CI: confidence interval
RFS: relapse-free survival
CSS: cancer-specific survival
CRP: C-reactive protein.
