# Supplementary material for: Comparison of the prognostic impact and combination of preoperative inflammation-based and/or nutritional markers in patients with stage II gastric cancer
Source: Oncotarget. 2018 Jun 29;9(50):29351–64. doi: 10.18632/oncotarget.25486 (PMC6047670; doi:10.18632/oncotarget.25486)
Supplement: Supplementary file 1 [file oncotarget-09-29351-s001.pdf]

Comparison of the prognostic impact and combination of preoperative inflammation-based and/or nutritional markers in patients with stage II gastric cancer

SUPPLEMENTARY MATERIALS

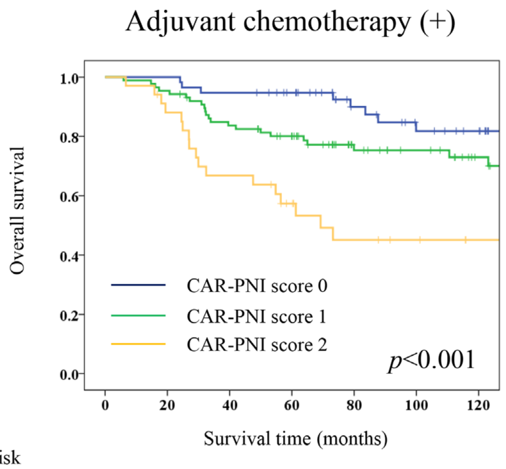

No. at risk

Score 0	57	57	54	49	35	28	24
Score 1	87	83	71	63	40	34	25
Score 2	34	29	22	16	11	9	6

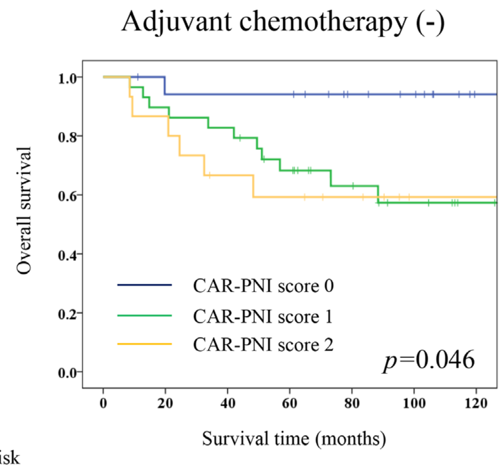

No. at risk

Score 0	18	16	16	16	11	9	2
Score 1	29	26	24	18	12	8	4
Score 2	15	13	9	8	6	2	2

Supplementary Figure 1: Kaplan-Meier survival curves of overall survival according to CAR-PNI scores in patients with adjuvant chemotherapy (p<0.001) and in patients without adjuvant chemotherapy (p=0.046).
